# Cardiac Gene Expression Knockdown Using Small Inhibitory RNA-Loaded Microbubbles and Ultrasound

**DOI:** 10.1371/journal.pone.0159751

**Published:** 2016-07-29

**Authors:** Jonathan A. Kopechek, Andrew R. Carson, Charles F. McTiernan, Xucai Chen, Edwin C. Klein, Flordeliza S. Villanueva

**Affiliations:** 1 Dept. of Medicine, University of Pittsburgh, Pittsburgh, PA, United States of America; 2 Dept. of Bioengineering, University of Louisville, Louisville, KY, United States of America; 3 Dept. of Laboratory Animal Resources, University of Pittsburgh, Pittsburgh, PA, United States of America; Swedish Neuroscience Institute, UNITED STATES

## Abstract

RNA interference has potential therapeutic value for cardiac disease, but targeted delivery of interfering RNA is a challenge. Custom designed microbubbles, in conjunction with ultrasound, can deliver small inhibitory RNA to target tissues *in vivo*. The efficacy of cardiac RNA interference using a microbubble-ultrasound theranostic platform has not been demonstrated *in vivo*. Therefore, our objective was to test the hypothesis that custom designed microbubbles and ultrasound can mediate effective delivery of small inhibitory RNA to the heart. Microbubble and ultrasound mediated cardiac RNA interference was tested in transgenic mice displaying cardiac-restricted luciferase expression. Luciferase expression was assayed in select tissues of untreated mice (n = 14). Mice received intravenous infusion of cationic microbubbles bearing small inhibitory RNA directed against luciferase (n = 9) or control RNA (n = 8) during intermittent cardiac-directed ultrasound at mechanical index of 1.6. Simultaneous echocardiography in a separate group of mice (n = 3) confirmed microbubble destruction and replenishment during treatment. Three days post treatment, cardiac luciferase messenger RNA and protein levels were significantly lower in ultrasound-treated mice receiving microbubbles loaded with small inhibitory RNA directed against luciferase compared to mice receiving microbubbles bearing control RNA (23±7% and 33±7% of control mice, *p*<0.01 and *p* = 0.03, respectively). Passive cavitation detection focused on the heart confirmed that insonification resulted in inertial cavitation. In conclusion, small inhibitory RNA-loaded microbubbles and ultrasound directed at the heart significantly reduced the expression of a reporter gene. Ultrasound-targeted destruction of RNA-loaded microbubbles may be an effective image-guided strategy for therapeutic RNA interference in cardiac disease.

## Introduction

RNA interference (RNAi) is a highly conserved endogenous cellular process whereby small interfering RNAs (siRNA) regulate post transcriptional gene expression via sequence-specific mRNA degradation and translational blocking. Due to its potent specific gene knockdown effects and efficiency, RNAi has emerged as a promising approach to treat molecular targets in a spectrum of human disease, including cardiac disease [1], but clinical translation has been stymied by inefficient delivery vectors and techniques. Viral vectors have been successful in mediating RNAi in cardiac tissues [2], but are limited by adverse off-target effects [3]. The ideal delivery vehicle should be targeted solely to the intended site of treatment and carry the therapeutic in a non-viral construct, thus reducing side effects while maintaining high levels of gene knockdown.

Custom designed microbubbles (MBs) are emerging as gene therapy vectors that may overcome several limitations of current RNAi delivery platforms and also function as true theranostic agents [4–6]. MBs consist of gas-filled microspheres encapsulated within a shell composed of phospholipids, polymers, or proteins and are generally 1–5 μm in diameter. MBs are routinely utilized in clinical cardiology as ultrasound contrast agents during echocardiography [7, 8]. The unique properties that make MBs ideal ultrasound contrast agents for echocardiography–intravascular kinetics comparable to that of erythrocytes and MB expansion and contraction in response to ultrasound–also confer capabilities for targeted molecular therapeutics [9, 10]. When exposed to appropriately tuned ultrasound, nucleic acid loaded MBs can be disrupted at desired target locations, releasing their payload, and transiently opening pores in neighboring cells to facilitate non-endosomal uptake of the nucleic acids (sonoporation) [11, 12]. Prior studies have found that adverse bioeffects of ultrasound targeted microbubble destruction in the myocardium are minor [13]. Ultrasound targeted MB destruction (UTMD) has been shown by us and others to deliver nucleic acids to cells and tissues *in vitro* and *in vivo* [5, 14–17].

We tested the hypothesis that a non-invasive and non-viral nucleic acid delivery platform utilizing MBs and ultrasound could mediate RNAi in the heart. To prove this concept, we used a customized MB formulation and acoustic regimen for cardiac-specific luciferase siRNA delivery in a transgenic mouse with cardiac-restricted expression of luciferase. Cardiac expression of luciferase was assayed at the RNA and protein levels.

## Methods

### Transgenic Mice

**Fig 1:**
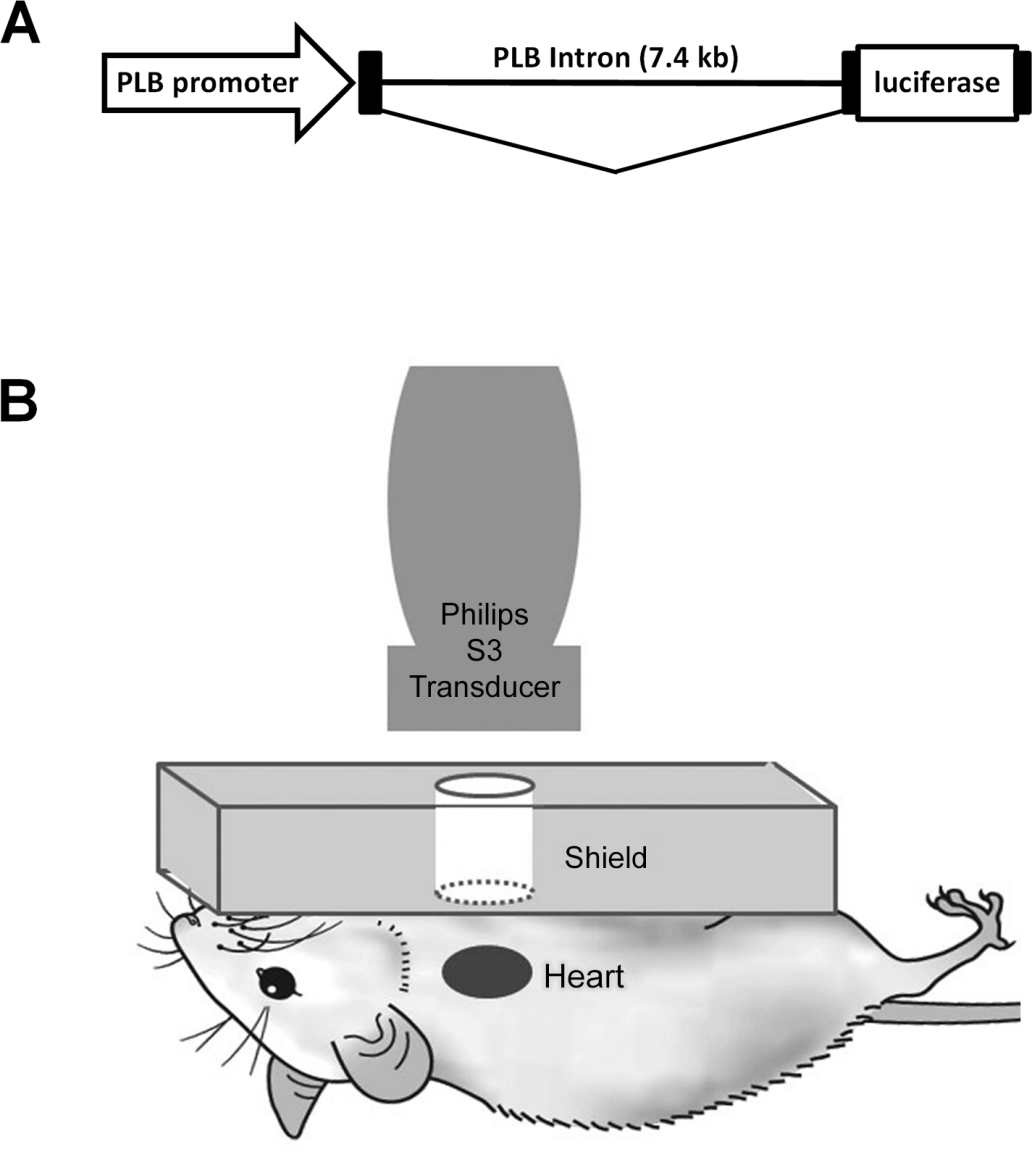
Experimental Design. (A) Schematic of rat phospholamban promoter/luciferase gene fusion used to generate transgenic mice. (B) Transducer and shield arrangement for cardiac delivery in siRNA *in vivo*. All elements are acoustically coupled using ultrasound gel.

Transgenic mice with cardiac-restricted expression of luciferase were produced by a commercial vendor through microinjection of the luciferase transgene into fertilized mouse egg nuclei (Chrysalis; DNX Transgenic Services) which was maintained in FVB (Charles River Laboratories) mice. Fig 1A illustrates the construction of the phospholamban promoter driven luciferase gene [18] used for the generation of transgenic mice. The luciferase transgene 2 contains ~11.5 kb of the rat phospholamban gene (4 kb upstream of transcription start site and 7.4 kb intron) with the rat phospholamban coding sequence replaced with firefly luciferase coding sequence (see reference [17] and Fig 1A for construct map). This promoter element has been demonstrated to drive luciferase expression to high levels in cardiomyocytes [18, 19]. Transgenic progeny were identified by routine PCR methods [20], using transgene-specific primers (5’-CGAGTGCTAACATATGAGAGGAG; 5’-CTTTATGTTTTTGGCGTCTTCCA).

### Microbubble preparation

Perfluorobutane gas-filled lipid MBs carrying either Silencer Select siRNA (Ambion) against luciferase or a negative control sequence were prepared as previously described [16, 17]. Briefly, chloroform solutions of 1,2-distearoyl-sn-glycero-3-phosphocholine, 1,2-distearoyl-sn-glycero-3-ethylphosphocholine, 1,2-distearoyl-sn-glycero-3-phosphoglycerol (Avanti Lipids), and polyethylene glycol-40 stearate (Sigma) at a mole ratio 100:43:1:4.5 were dried with argon gas. An aqueous micellar solution of lipid was then prepared by adding phosphate buffered saline (PBS) containing 1 mmol/L EDTA and the solution was sonicated (Misonix) to disperse lipids. The resulting lipid solution was diluted 1:4 in PBS/EDTA, and 8 μg of Silencer Select siRNA against luciferase or a negative control sequence was added. The solution was placed in a sealed glass vial and the head space was filled with perfluorobutane gas (FlouroMed), followed by amalgamation (Bristol Myers-Squibb) to form perfluorobutane gas-filled MBs [17]. The resulting siRNA-loaded MB solution contained approximately 7×10^8^ MB/mL with a mean diameter of 2.1±1.1 μm as measured by a Coulter Multisizer 3 (Beckman Coulter).

### Animal ethics statement

All animal procedures conformed to NIH guidelines for the care and use of laboratory animals and were approved by the Institutional Animal Care and Use Committee at the University of Pittsburgh. For all surgical procedures, mice were anesthetized using 1–2% inhaled isoflurane and the depth of anesthesia was monitored by toe pinch response. All mice were euthanized by resection of the heart and decapitation under deep isoflurane anesthesia (5%).

### Microbubble and ultrasound treatment protocol

While mice were under anesthesia a venous cannula was surgically placed in the jugular vein for MB infusion. To shield the lungs from ultrasound, acoustic absorbing material with a circular window (10 mm diameter) overlying the heart was placed on the chest (Fig 1B), with cardiac alignment confirmed by B-mode ultrasound imaging using a transducer placed over the window (14 MHz, Sequoia 512, Siemens). Thereafter, the imaging probe was replaced with a second probe (1.3 MHz, Sonos 7500, Philips) to deliver therapeutic ultrasound (Fig 1B). The treatment protocol was first tested in 3 “mock” mice without the acoustic shield in place, which allowed for simultaneous contrast-specific imaging (Contrast Pulse Sequencing, 7 MHz, Sequoia 512, Siemens) of MB destruction and replenishment. This approach guided the selection of an ultrasound treatment regimen comprising 2 ultrasound bursts (frames) at a mechanical index (MI) of 1.6, repeated at 0.5 second intervals.

A 0.5–0.7 mL suspension of MBs loaded with either luciferase siRNA (n = 9) or negative control siRNA (n = 8) was infused over 15 minutes during simultaneous treatment with the ultrasound regime described above. Ultrasound treatment was continued for an additional 5 minutes after the end of the infusion (total ultrasound duration of 20 minutes). After treatment, the venous cannula was removed and the mice were recovered. Three days thereafter, the mice were euthanized as described above, and tissues were excised for measurement of luciferase mRNA and protein levels.

To determine if UTMD causes tissue injury, hearts and lungs were harvested from 3 additional mice 1.5 hours after UTMD treatment and fixed in formalin. As a negative control, the heart and lungs were also harvested from a single mouse that did not receive UTMD treatment. Tissue sections were stained with hematoxylin and eosin (H&E) and read by a veterinary pathologist blinded to experimental condition to identify any evidence of hemorrhage, petechiae, or other treatment-related injury such as necrosis.

### Luciferase detection

Ventricles were harvested post-mortem to quantify luciferase expression in 14 untreated mice in order to establish baseline levels of luciferase expression in target tissue. Tissue was collected from the atria, kidney, liver, lung, spleen and uterus in 5 of these 14 untreated mice to confirm cardiac restricted luciferase expression. All tissue samples were homogenized and protein content was estimated by Bradford assay. Luciferase activity was detected using a commercial assay (Promega) with a 20 second sampling time. Luciferase activity in treated mice was normalized to total protein and expressed as a percent of the mean luciferase activity detected in the ventricles of untreated mice. Data are reported as mean ± standard error.

### Real time quantitative PCR

Ventricles from 8 of 14 untreated mice were processed for real time PCR to establish baseline levels of luciferase mRNA expression. A subset of the ventricles from luciferase siRNA treated mice (n = 8), and control siRNA treated mice (n = 7) were processed for real time PCR. RNA was extracted from tissue samples using Trizol reagent (Invitrogen), and complementary DNA was prepared from 2 μg of total RNA using Taqman reverse transcription kit (Applied Biosystems). Luciferase expression was determined by using RT PCR primers (5′-TCTAAGGAAGTCGGGGAAGC; 5′-CCCTCGGGTGTAATCAGAAT). The GAPDH gene (5′-GGCAAATTCAACGGCACAGT; 5′-AGATGGTGATGGGCTTCCC) was used as a reference to normalize luciferase measurements. RT-PCR amplifications were performed with the Absolute blue Sybr Green/Rox kit (Thermo Scientific) using an Applied Biosystems 7900HT instrument.

### Acoustic cavitation detection

To further characterize the ultrasound cavitation phenomena associated with the treatment protocol, acoustic emissions from MB cavitation were recorded passively in a single separate mouse using a 3.5-MHz single-element transducer (V383, Olympus NDT) with a 25 mm focal depth, positioned 25 mm from the heart and aligned to the focus of the treatment transducer both prior to, and during, MB infusion. The acoustic shield was removed to facilitate the alignment of the transducer and passive cavitation detector over the mouse heart. Acoustic emission signals were obtained upon ultrasound delivery as described above (1.3 MHz, MI = 1.6) before and during MB infusion. All received acoustic signals were filtered using a 2-MHz high pass filter to reduce the 1.3-MHz fundamental frequency, and amplified by 20 dB using a pulser/receiver in receive mode (5073PR, Olympus NDT). Signals were digitized at a 25-MHz sampling frequency using a digital oscilloscope (WaveRunner 6051A, LeCroy) and processed in Matlab (Mathworks). An 8-microsecond time gate was applied to each signal and a Fourier transform was performed to the time-domain signals in order to obtain the frequency domain spectra.

### Statistical analysis

Luciferase activity in luciferase siRNA-MB or control siRNA-MB treated mouse ventricles was normalized to the average luciferase activity found at baseline in the ventricles of non-treated mice and reported as mean ± standard error. Analysis of luciferase expression by RT-PCR was performed using the 2^−ΔΔCt^ method using untreated mice as the calibrator, and expressed as mean ± standard error. Luciferase expression at the mRNA and protein levels was not normally distributed and therefore a Mann-Whitney test was used to perform statistical comparisons between groups.

## Results

### Characterization of luciferase expression in transgenic mice

**Fig 2:**
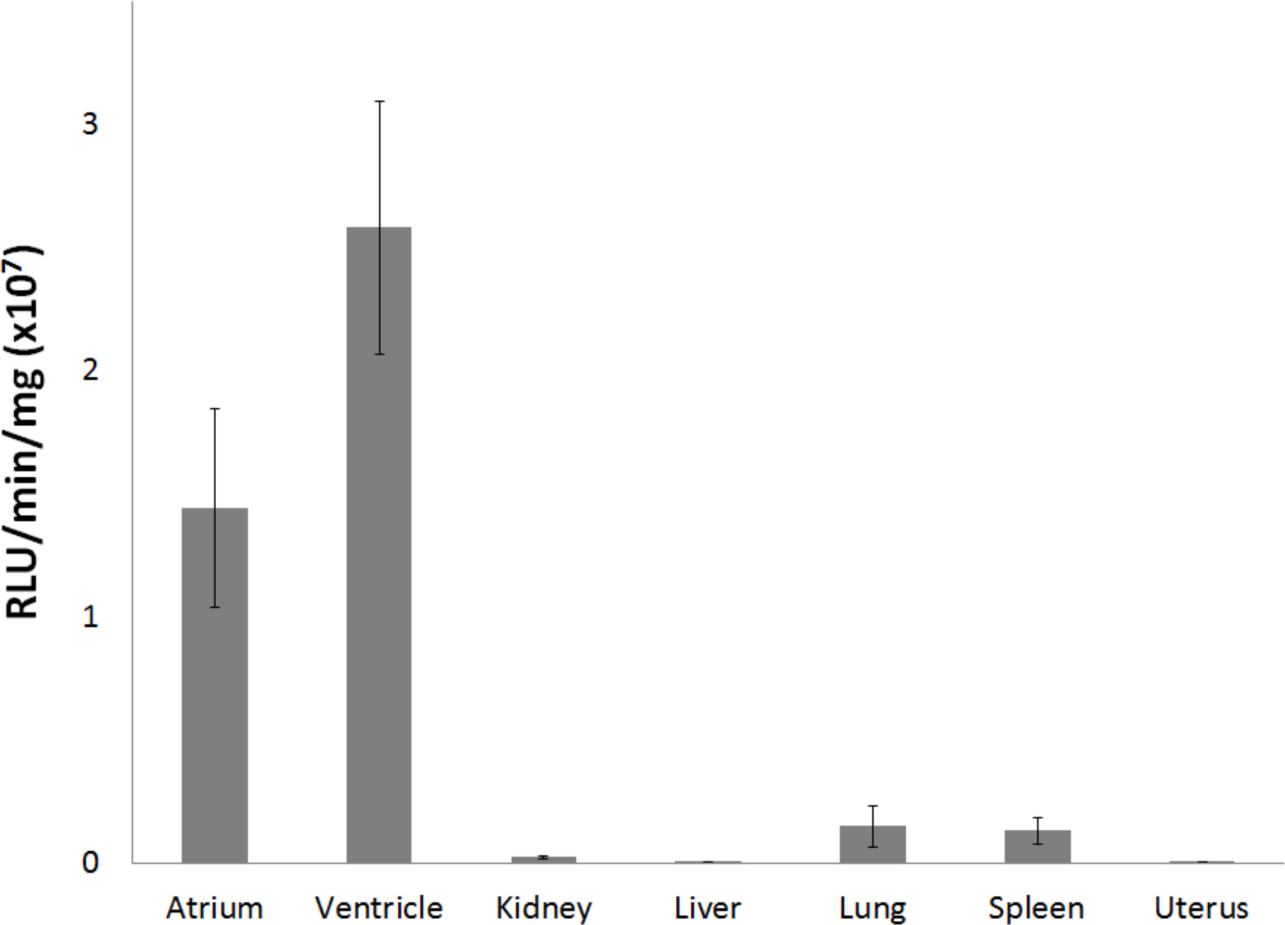
Characterization of luciferase transgenic mice. Average luciferase activity (±SE) of selected tissues obtained from PLB-Luc transgenic mice (n = 5).

Luciferase activity was measured in different organs to confirm whether expression was restricted to the heart. High levels of luciferase activity were detected in cardiac tissue, with minimal activity detected in the kidney, liver, lung, spleen, or uterus (Fig 2).

### Cardiac delivery of luciferase siRNA

**Fig 3:**
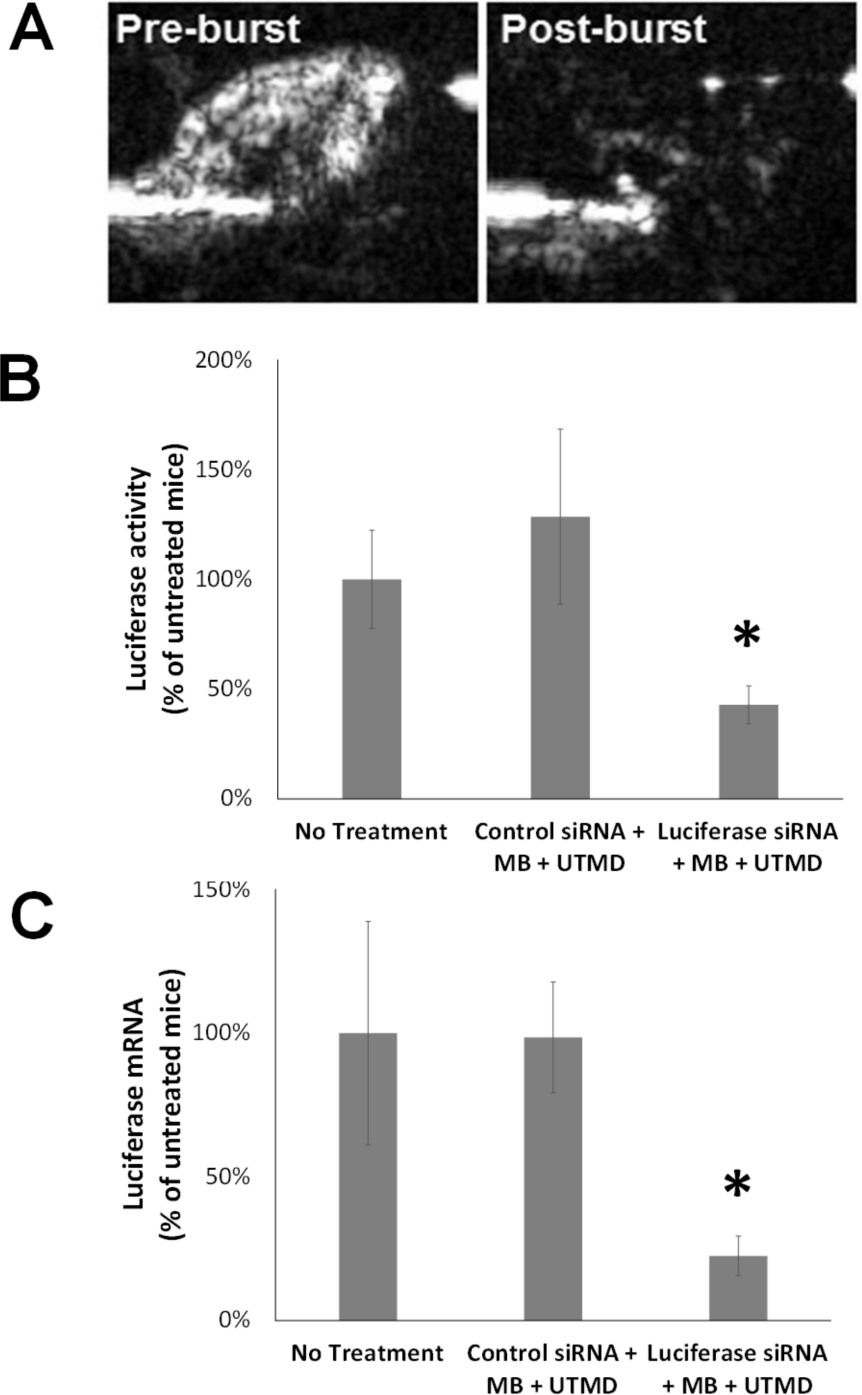
Ultrasound and Microbubble Delivery of siRNA to the Heart. Therapeutic ultrasound during transit of luciferase siRNA-loaded MB through the heart causes microbubble destruction and reduces cardiac luciferase expression. (A) Contrast pulse sequence imaging of mouse heart before (pre-burst) and after (post-burst) UTMD. (B) Normalized luciferase activity (±SE) in the ventricles of mice 3 days after intravenous delivery of negative control siRNA-loaded MBs (NC-MB) + UTMD (n = 8) or luciferase siRNA-loaded MB (Luc-MB) + UTMD (n = 9). (C) Normalized luciferase mRNA levels (±SE), as assayed by RT-PCR, in the ventricles of mice 3 days after delivery of negative control siRNA-loaded MB (NC-MB) + UTMD (n = 7) or luciferase siRNA-loaded MB (Luc-MB) + UTMD (n = 8). * indicates significant differences between treated groups (*p*<0.05) by Mann-Whitney test.

Ultrasound targeted microbubble destruction was observed in mouse hearts following infusion of luciferase-loaded MBs, as shown in Fig 3A. There was homogeneous opacification of the heart prior to ultrasound treatment, and complete MB destruction immediately following the therapeutic ultrasound pulse.

Three days after treatment, normalized luciferase protein activity was significantly reduced (by over 60%) in the ventricles of mice treated with luciferase siRNA-loaded MBs compared to ventricles of mice treated with control siRNA-loaded MBs (*p* = 0.03, Fig 3B). Similarly, normalized ventricular luciferase expression at the mRNA level was significantly less (by about 70%) in mice treated with luciferase siRNA-loaded MBs than in mice treated with control siRNA-loaded MBs (*p*<0.01, Fig 3C).

**Fig 4:**
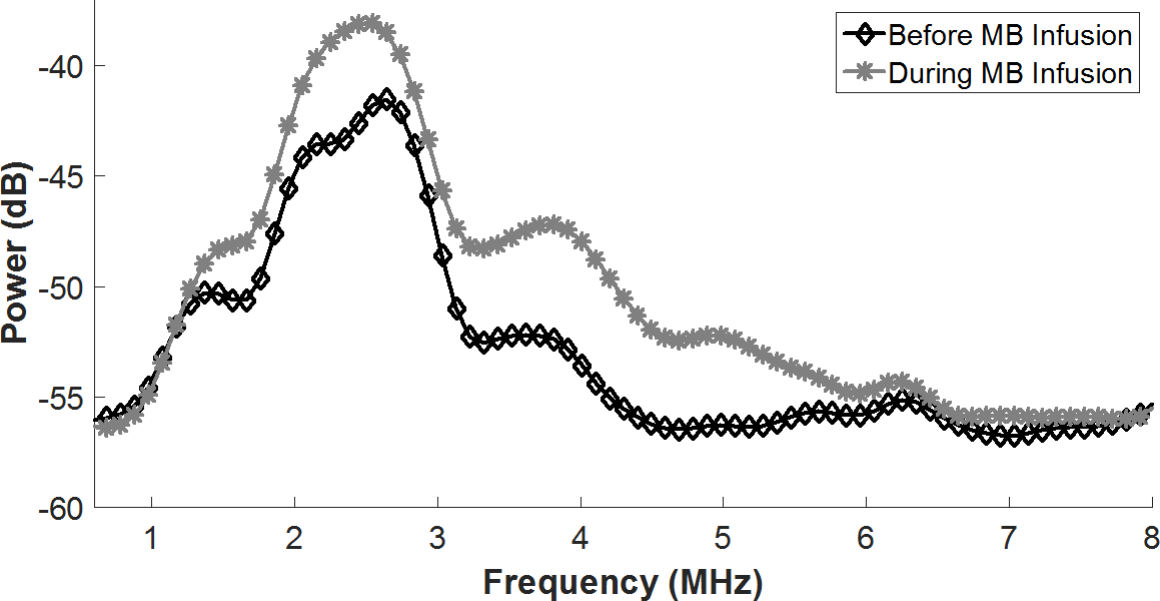
Microbubble Cavitation in Mouse Heart. Average acoustic emissions in a single, representative mouse upon ultrasound delivery before (◊) and during (•) MB infusion. Broadband increase in average acoustic emissions during MB infusion indicates the presence of MB inertial cavitation.

Passive cavitation detection performed before and during MB infusion and therapeutic ultrasound revealed increased broadband emissions upon MB infusion (Fig 4), which indicate inertial cavitation behavior of the MBs in response to the therapeutic ultrasound pulses.

### Histological assessment of UTMD treatment-related injury

**Fig 5:**
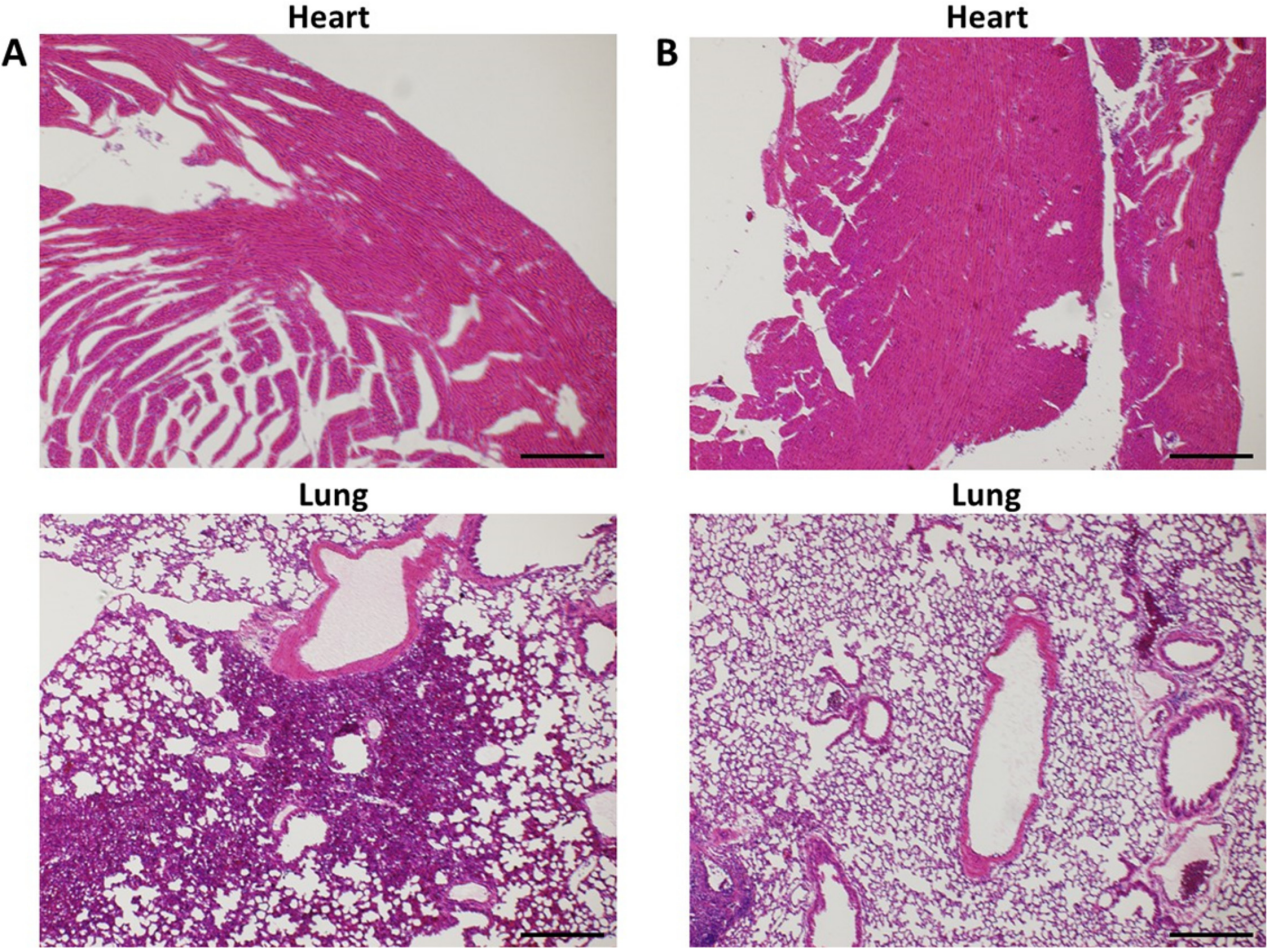
Representative H&E Images of Mice Hearts and Lungs Post-UTMD Treatment. (A) Representative H&E images of mouse heart and lung following UTMD treatment with evidence of focal lung hemorrhage. (B) Representative H&E images of mouse heart and lung following UTMD treatment with no evidence of focal lung hemorrhage. Scale bars represent 100 μM.

The excised heart and lung blocs appeared grossly normal, with no visual evidence of hemorrhage. H&E stained myocardial tissue sections were entirely normal, and there was no histologic evidence of myocardial injury or inflammation. There was a single focus of microscopic lung hemorrhage (approximate area of 0.05 mm^2^) in 2 of 3 mice following UTMD treatment, falling within an area outside of our acoustic shield. There was no clinical evidence of respiratory distress following UTMD treatment. Representative images are shown in Fig 5.

## Discussion

Our primary finding is that custom designed siRNA-loaded MB, in conjunction with ultrasound parameters inducing inertial cavitation, deliver siRNA to murine heart tissue, resulting in reduced expression of a reporter gene. The degree of reporter gene silencing was substantial at both the mRNA and protein levels, and this finding establishes proof of concept that this intravascular delivery platform containing only 8 micrograms siRNA can impact cardiac biology. To our knowledge, this is the first report of systemic intravenous injection of siRNA-loaded MBs and ultrasound to functionally deliver siRNA to the heart.

There have been previous descriptions of UTMB to deliver nucleic acids to the heart: A prior study demonstrated that an invasive intra-LV co-injection of microbubbles and 40 micrograms siRNA, when followed by ultrasound treatment, could result in target gene knockdown predominantly in heart vasculature with little effect on cardiomyocytes [21]. A recent study reported successful delivery of miRNA interference plasmid to the heart following a complex series of 6 systemic co-injections of 2 milligrams plasmid DNA and microbubbles and ultrasound treatments [22]. In this system, treatment effects were detected in cardiomyocytes, indicating a similar delivery platform can reach cardiomyocytes, although delivery of plasmid DNA may face different challenges. Lentiviral vectors have also been delivered to cells using ultrasound and microbubbles and offer an alternative approach to induce siRNA expression in targets cells [23]. However, delivery of siRNA directly should enable a more rapid therapeutic effect compared to delivering lentiviral vectors expressing siRNA. Importantly, UTMD-mediated delivery of siRNA loaded on microbubbles avoids reliance on a viral vector. Our report is distinct from these prior studies in that we describe a non-viral, non-invasive (intravenous injection), non-plasmid method for direct delivery of siRNA to the myocardium. As such, our study has promising implications for clinical translation of siRNA-based treatments for cardiac disease.

Although RNA interference technology has shown great potential in preclinical applications [24] *in vivo* delivery of siRNA molecules is challenged by their susceptibility to nucleases and renal excretion [24]. The use of MBs as siRNA vectors has several unique advantages. We have previously shown that MBs shield siRNA from nucleases [17], and at >2μm in diameter, are too large for renal excretion. With UTMD, intravenously injected MBs are disrupted by ultrasound as they pass through the microcirculation, resulting in perivascular deposition of nucleic acids without significant microvascular hemorrhage [25]. Although the mechanisms underlying the efficacy of UTMD are incompletely understood, several observations have shed light: Ultrasound induced vibrations of MBs touching cell membranes increases membrane permeability *in vivo* and *in vitro* [11, 26]. UTMD pulses similar to those used in this study can produce high local pressure currents (jetting) and/or high velocity fragmentation of the MB shell, which may also increase membrane permeability [27]. These phenomena may contribute to the sonoporation event whereby transient pores are opened in target cell membranes [28], allowing influx of therapeutic molecules, such as siRNA, into the cytoplasm. This mechanism enables spatial targeting, selective uptake, and trafficking to the cytoplasm in a single noninvasive step. Lastly, because MBs are ultrasound contrast agents, the distribution and delivery of the therapeutic can be imaged and adjusted during treatment, allowing real time image-guided siRNA delivery.

Ultrasound at higher intensities is scattered by air in the lungs and this could produce pulmonary vascular hemorrhage, which could potentially be monitored with computed tomography [29]. No gross or histologic evidence of injury was observed in mouse hearts following UTMD treatment. However, a single focus of microscopic lung hemorrhage was observed in 2 of 3 separately studied mice 1.5 hours after UTMD treatment, although the hemorrhagic area was small (0.05 mm^2^) and no clinical signs of respiratory distress were observed. Because the ultrasound beam used in this study encompassed the entire chest of the mice, a protective acoustic absorbing shield was fashioned, but will be less important in clinical applications, where the beam elevation is small relative to the size of the heart and ultrasound exposure of the lungs can be more easily avoided.

Several limitations to this study bear mention. We cannot definitively conclude that the luciferase silencing phenomenon observed in this study derived solely from the cardiomyocytes, as other cell types within the heart could have also expressed luciferase, such that the luciferase silencing that was measured could have been partially derived from these cell types. However, the phospholamban expression cassette used in this animal model has been shown to drive luciferase expression to high levels in cardiomyocytes *in vitro* [18], and we thus infer that the luciferase gene silencing we observed was predominantly attributable to cardiomyocyte luciferase gene expression knockdown. The spatial targeting of gene expression knockdown achieved by our platform is not definitively demonstrated here, as luciferase expression was by design restricted to the heart in this animal model.

The duration of luciferase knockdown following siRNA delivery was not assessed in this study. Previous *in vivo* studies found that siRNA reduced luciferase expression in mice for up to 10 days in tumor cells and up to 3–4 weeks in non-dividing hepatocytes [30, 31]. This suggests that UTMD-mediated delivery of siRNA to non-dividing cells such as cardiomyocytes may persist for up to several weeks but further studies are warranted to explore this topic. Finally, we chose a reporter gene, and not a clinically relevant gene, to demonstrate the efficacy of our ultrasound/MB platform. This was a deliberate choice in order to facilitate critical proof of concept without the confounding influences of cardiac pathology.

Ultimately, UTMD could have therapeutic value in cardiac pathophysiologic states for which non-druggable gene targets have been identified. For example, other preclinical studies have previously demonstrated siRNA-mediated protection of the heart following myocardial infarction or ischemia/reperfusion injury by targeting genes such as Nox2 and HIF-1alpha-prolyl-4 hydroxylase-2, although non-invasive targeted delivery approaches were not employed [32, 33]. Importantly, our findings can now justify further studies to confirm similar effects of our ultrasound-microbubble delivery platform on natural genes in diseased myocardium.

In conclusion, MBs and ultrasound can deliver siRNA to the heart, resulting in gene expression knockdown. The use of a direct intravenous injection and application of external ultrasound is minimally invasive, inherently image-guided by virtue of ultrasound-enabled imaging and treatment, and amenable to serial treatments [16, 17]. This platform technology could be particularly attractive in reducing the expression of specific genes associated with cardiac disease, especially those genes that have not or cannot be targeted by conventional pharmaceuticals.

## Supporting Information

S1 Dataset Characterization of luciferase transgenic mice. (XLSX)

S2 Dataset Ultrasound and Microbubble Delivery of siRNA to the Heart. (XLSX)

S3 Dataset Microbubble Cavitation in Mouse Heart. (XLSX)

S4 Dataset Representative H&E Images of Mice Hearts and Lungs Post-UTMD Treatment. (ZIP)
