# Supplementary figures and images for: Cardiac Gene Expression Knockdown Using Small Inhibitory RNA-Loaded Microbubbles and Ultrasound
Source: PLoS One. 2016 Jul 29;11(7):e0159751. doi: 10.1371/journal.pone.0159751 (PMC4966949; doi:10.1371/journal.pone.0159751)

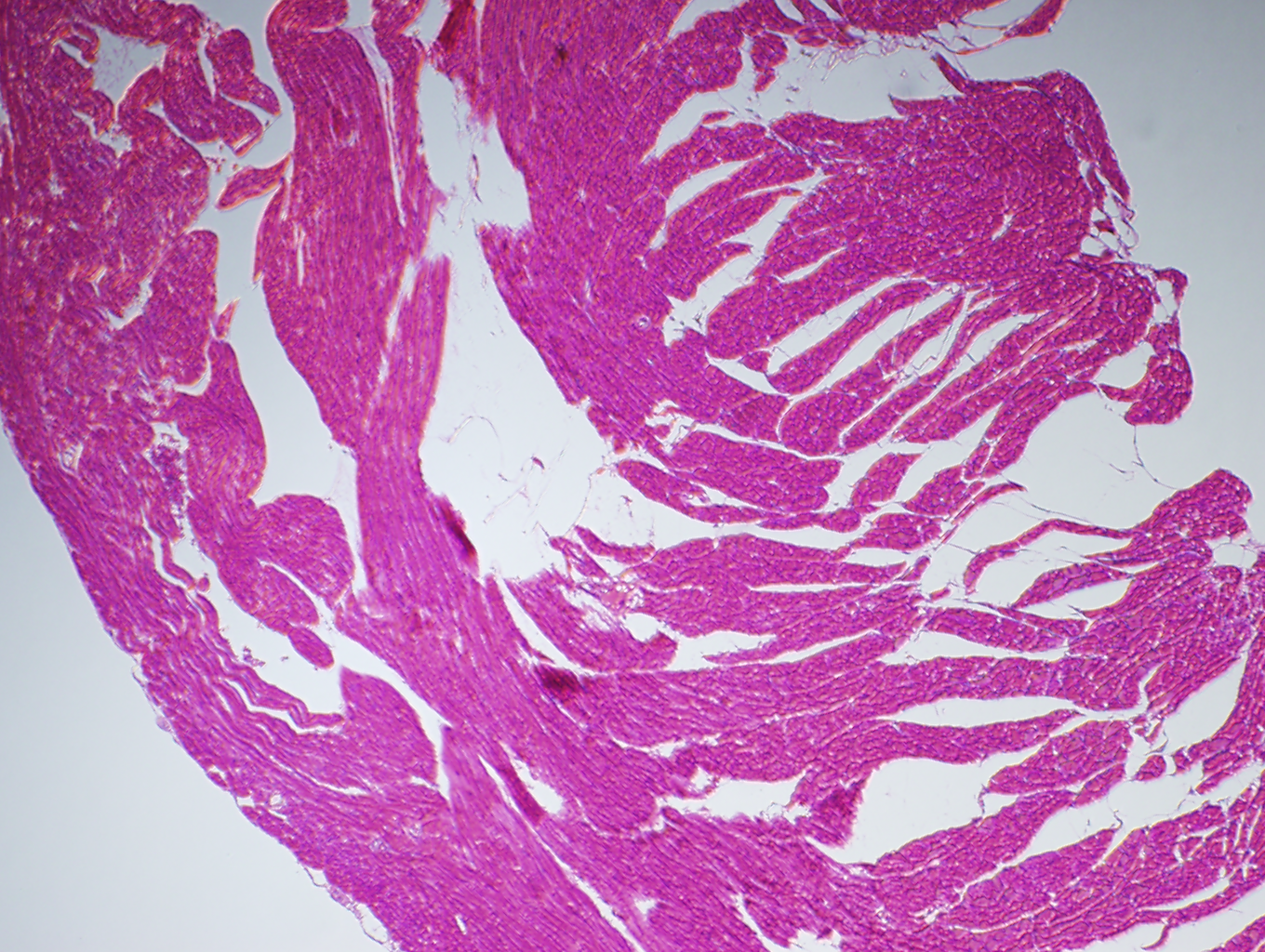

Supplement: S4 Dataset — (ZIP) [file pone.0159751.s004.zip › HandE_Microscopy_Images/NT_Control_heart.tif]

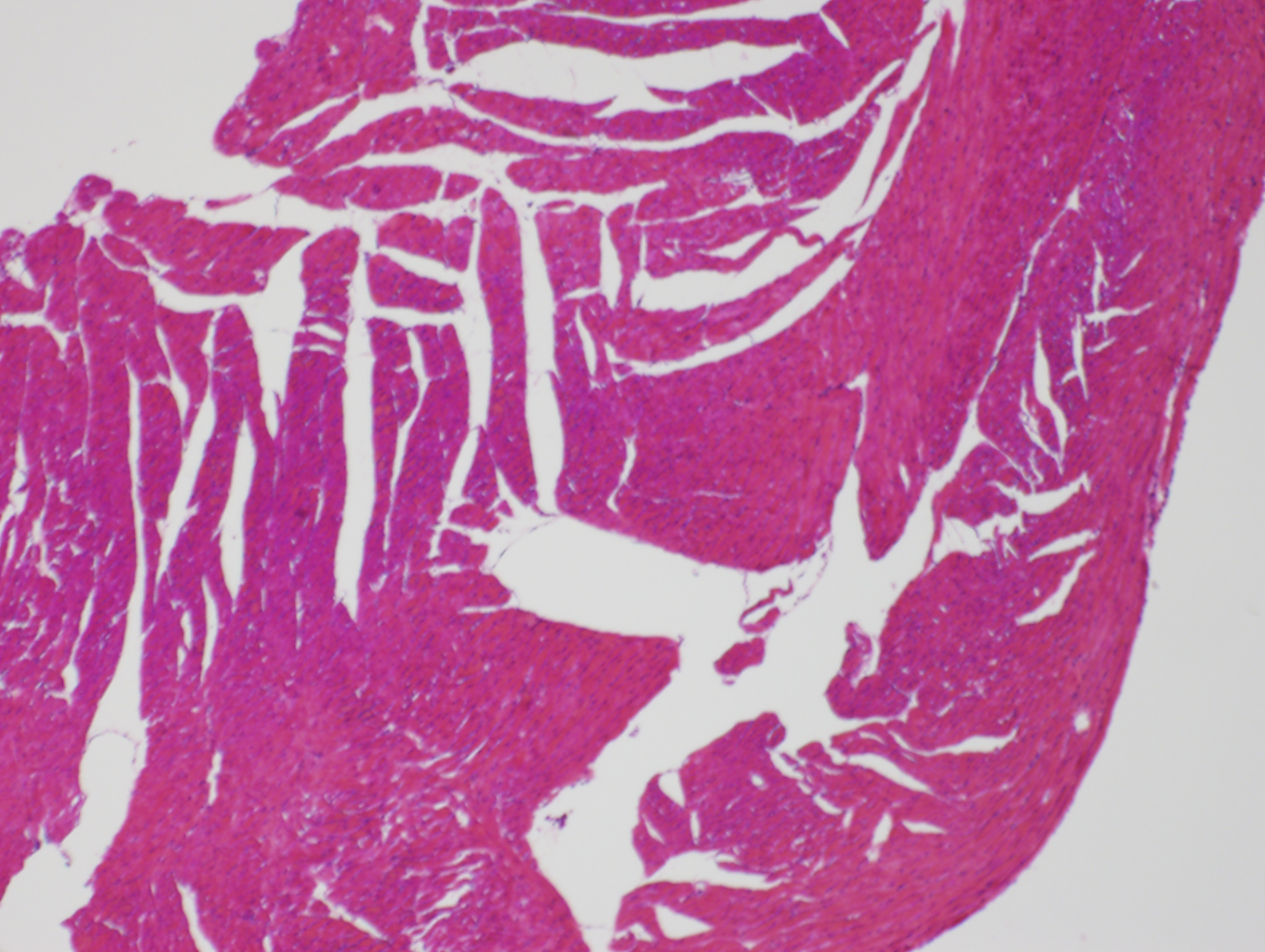

Supplement: S4 Dataset — (ZIP) [file pone.0159751.s004.zip › HandE_Microscopy_Images/siRNA_UTMD_mouse1_heart.tif]

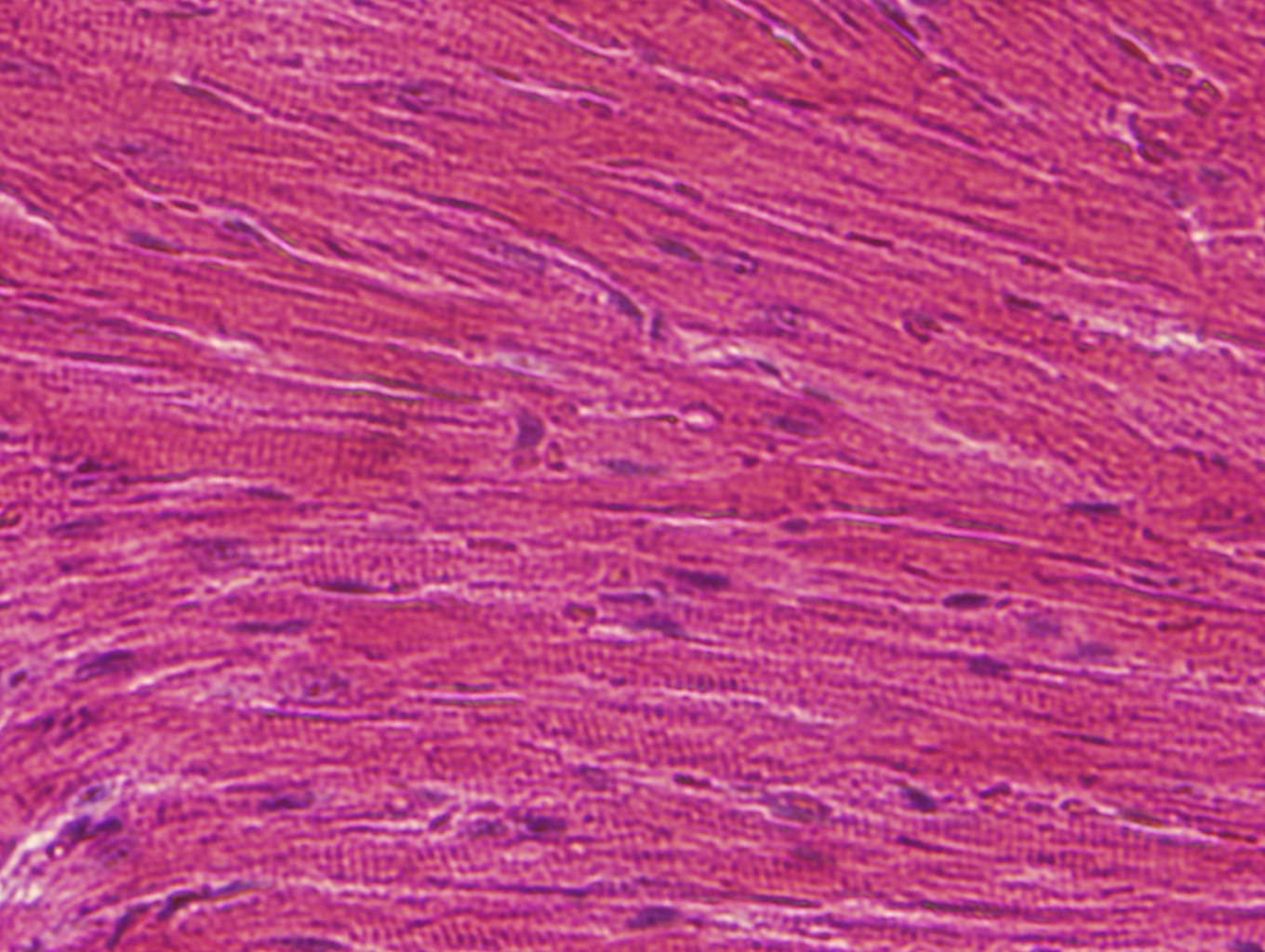

Supplement: S4 Dataset — (ZIP) [file pone.0159751.s004.zip › HandE_Microscopy_Images/siRNA_UTMD_mouse2_heart_40x.tif]

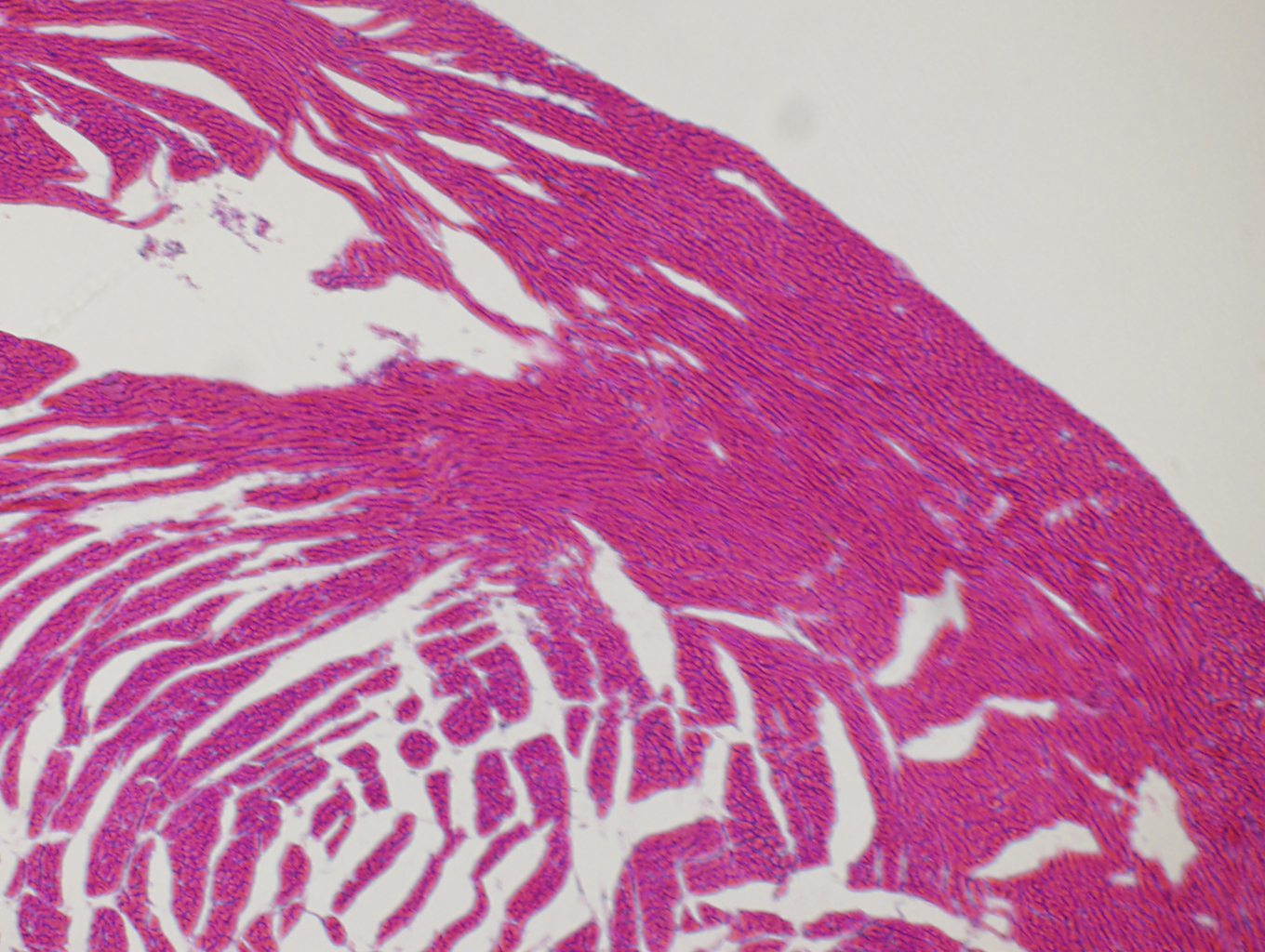

Supplement: S4 Dataset — (ZIP) [file pone.0159751.s004.zip › HandE_Microscopy_Images/siRNA_UTMD_mouse2_heart_4x.tif]

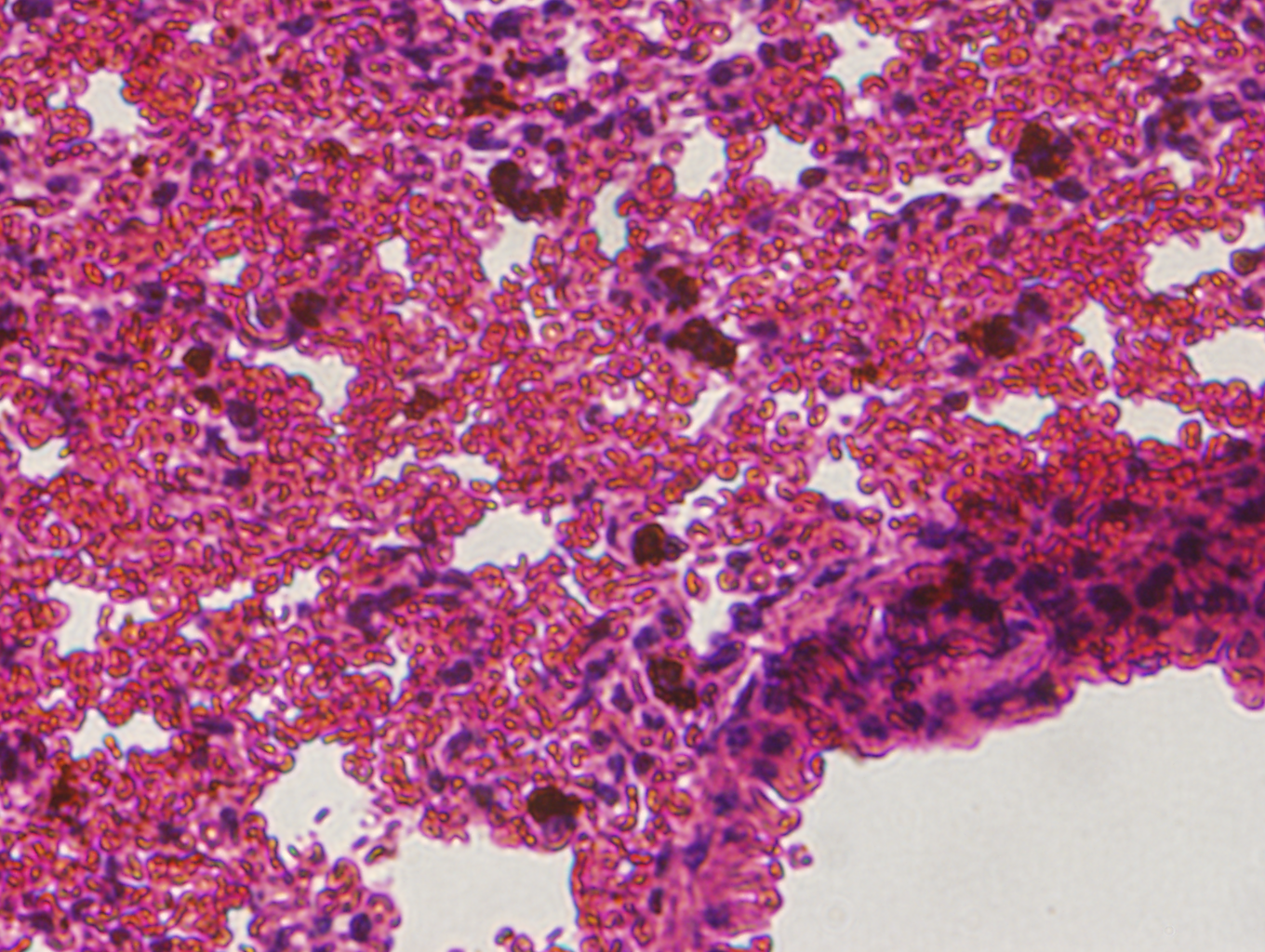

Supplement: S4 Dataset — (ZIP) [file pone.0159751.s004.zip › HandE_Microscopy_Images/siRNA_UTMD_mouse2_lung_40x.tif]

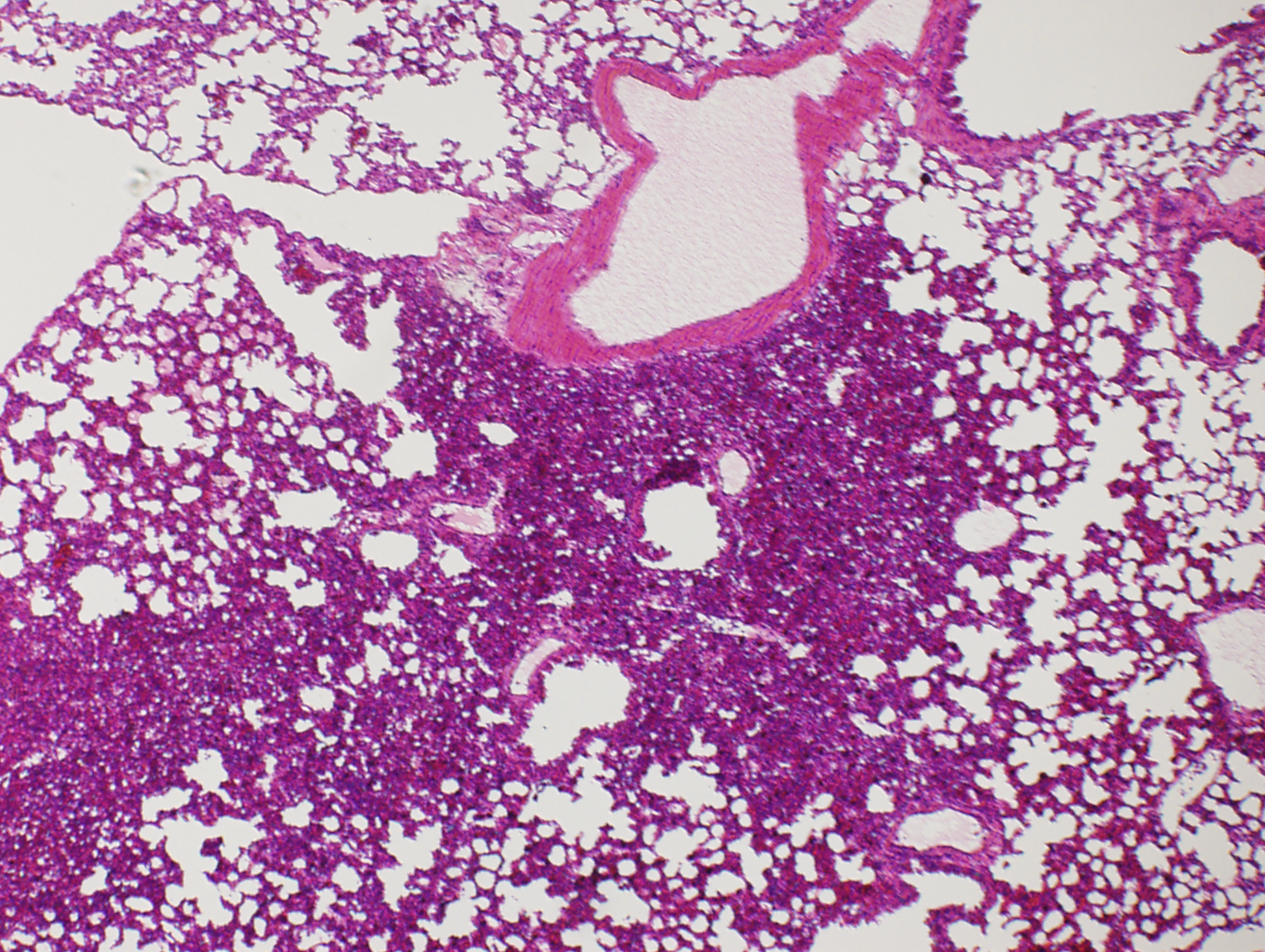

Supplement: S4 Dataset — (ZIP) [file pone.0159751.s004.zip › HandE_Microscopy_Images/siRNA_UTMD_mouse2_lung_4x.tif]

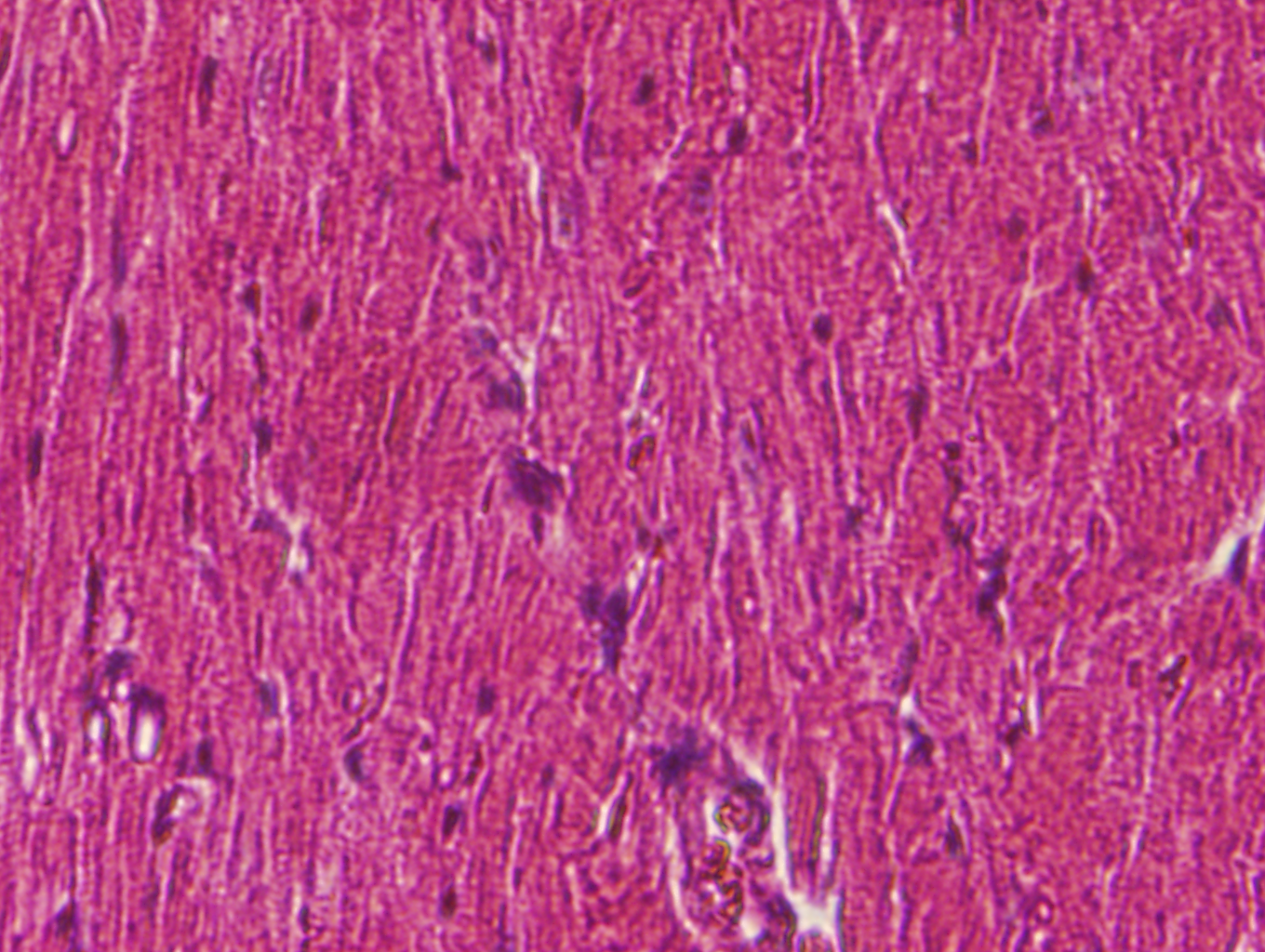

Supplement: S4 Dataset — (ZIP) [file pone.0159751.s004.zip › HandE_Microscopy_Images/siRNA_UTMD_mouse3_heart_40x.tif]

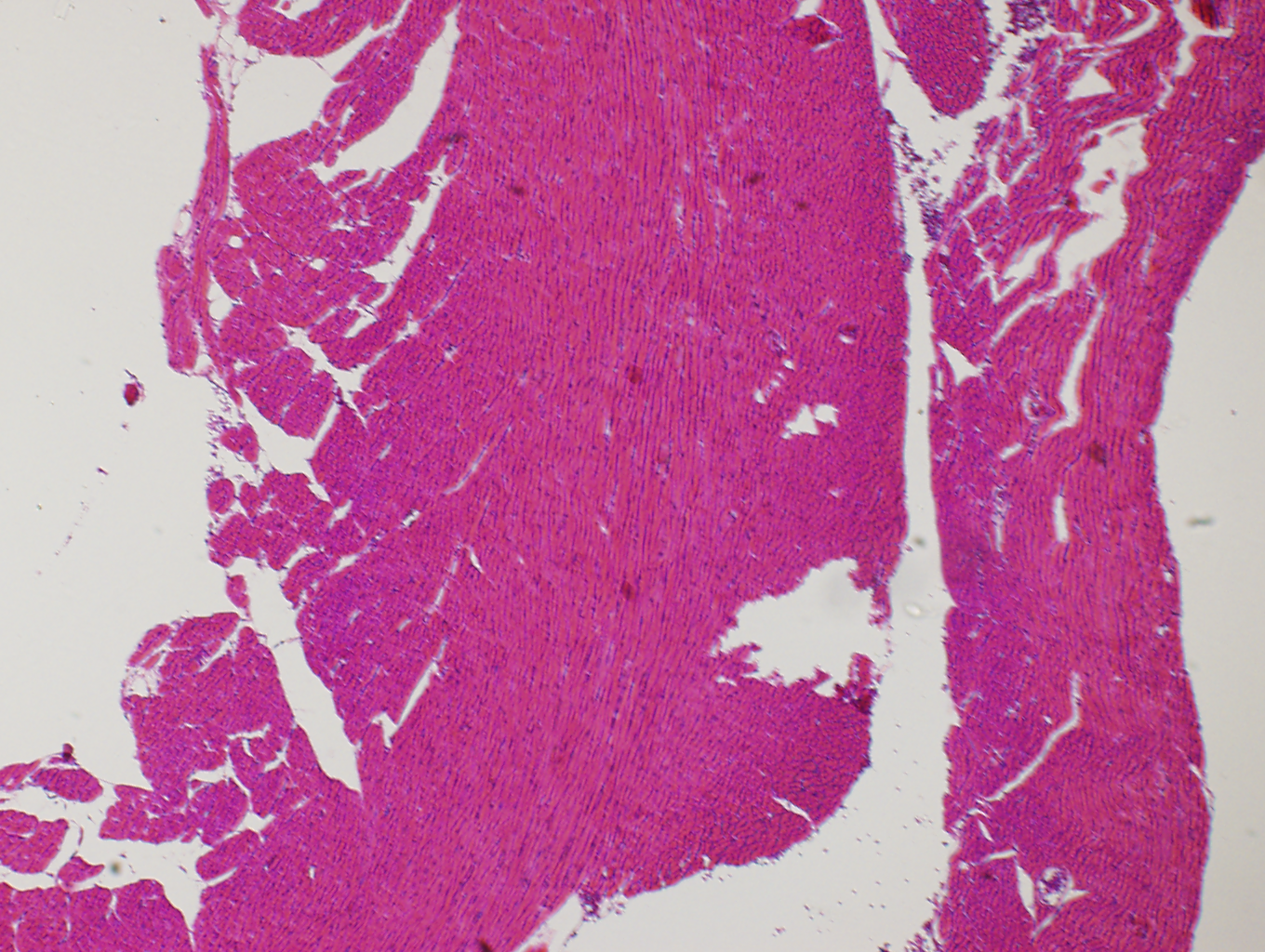

Supplement: S4 Dataset — (ZIP) [file pone.0159751.s004.zip › HandE_Microscopy_Images/siRNA_UTMD_mouse3_heart_4x.tif]

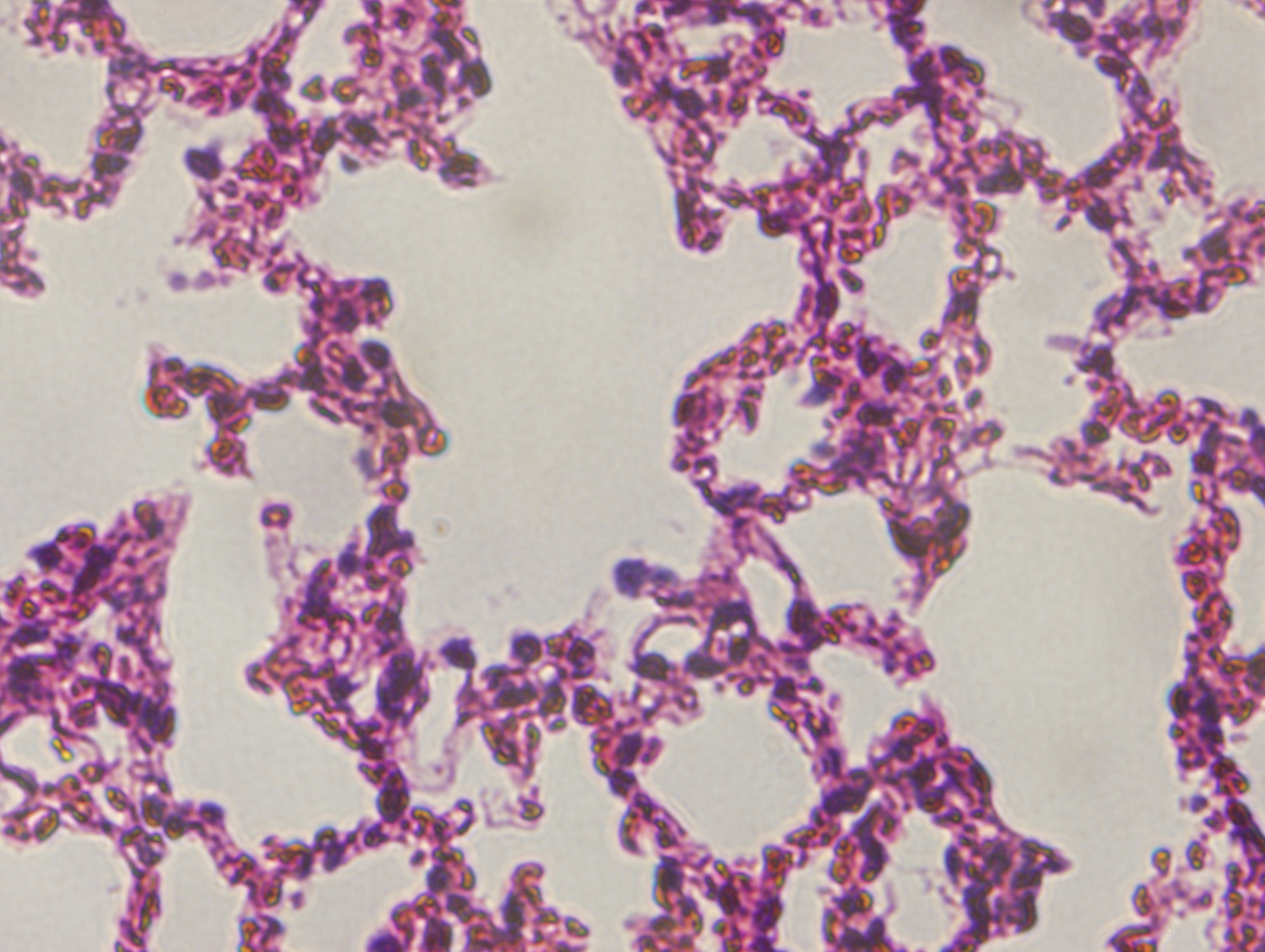

Supplement: S4 Dataset — (ZIP) [file pone.0159751.s004.zip › HandE_Microscopy_Images/siRNA_UTMD_mouse3_lung_40x.tif]

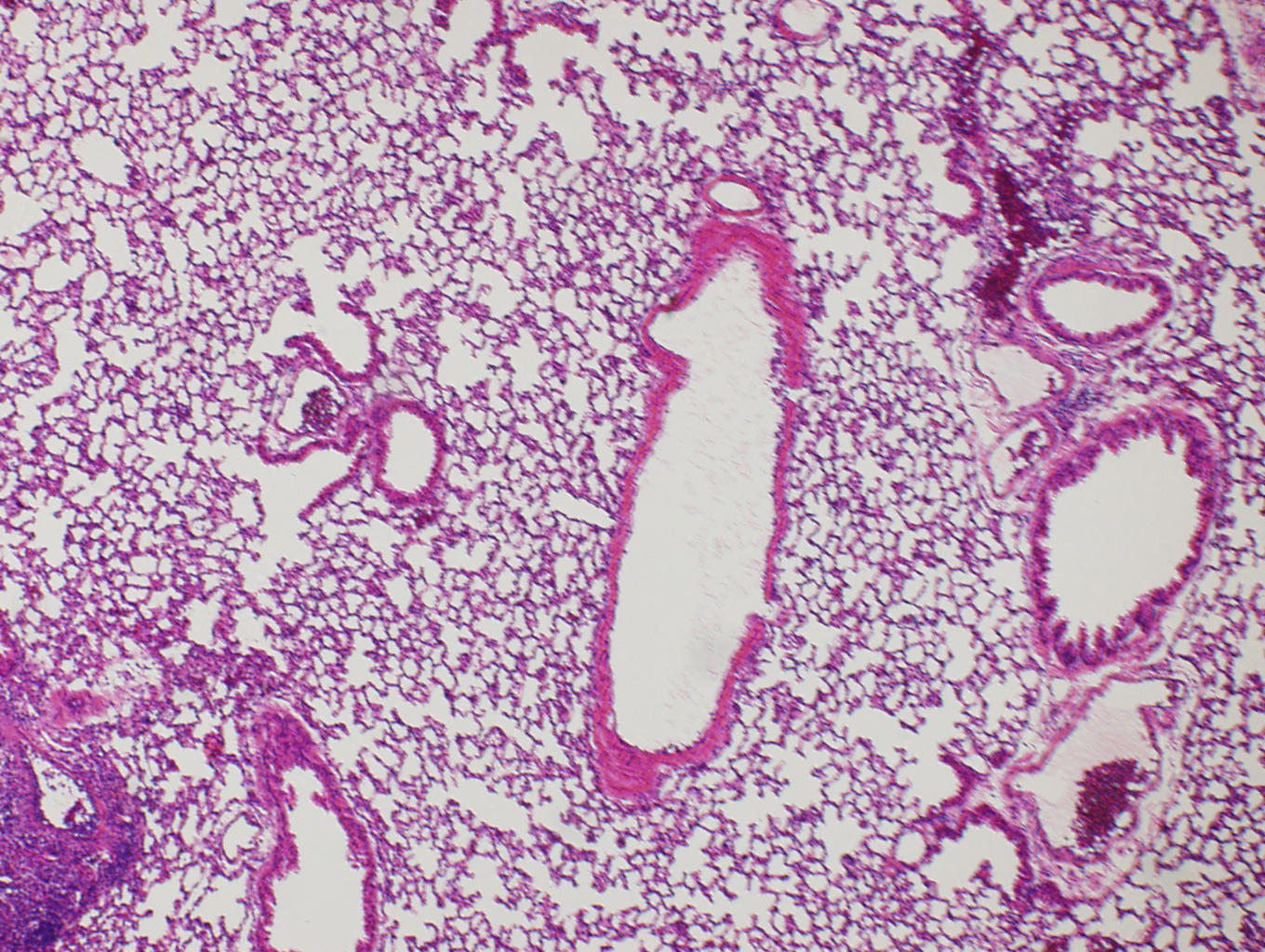

Supplement: S4 Dataset — (ZIP) [file pone.0159751.s004.zip › HandE_Microscopy_Images/siRNA_UTMD_mouse3_lung_4x.tif]
